# *catena*-Poly[hemi{bis­[4′-(3-pyrid­yl)-2,2′:6′,2′′-terpyridine-κ^3^ *N* ^1^,*N* ^1′^,*N* ^1′′^]copper(II)} [cuprate(I)-di-μ_2_-thio­cyanato-κ^2^ *N*:*S*;κ^2^ *S*:*N*]]

**DOI:** 10.1107/S1600536809023356

**Published:** 2009-06-24

**Authors:** Wen-Juan Shi

**Affiliations:** a Jiangxi Key Laboratory of Surface Engineering, Jiangxi Science and Technology Normal University, Jiangxi 330013, People’s Republic of China

## Abstract

The title compound, {[Cu(C_20_H_14_N_4_)_2_][Cu_2_(NCS)_4_]}_*n*_, was obtained by reacting copper acetate hydrate, ammonium thio­cyanate and 4′-(3-pyrid­yl)-2,2′:6′,2′′-terpyridine (3-pytpy) under solvothermal conditions. The polymeric complex is isostructural with the 4′-phenyl-2,2′:6′,2′′-terpyridine (phtpy) analogue. All intramolecular distances and angles are very similar for the two structures. Substitution of a phenyl group with a pyridyl group has no significant effect on the crystal packing which is accomplished by C—H⋯N and C—H⋯S hydrogen-bonding interactions.

## Related literature

For background to 2,2′:6′,2′′-terpyridine derivatives and their complexes, see: Andres & Schubert (2004 ▶); Constable (1986 ▶); Hofmeier & Schubert (2004 ▶). For the isostructural 4′-phenyl-2,2′:6′,2′′-terpyridine (phtpy) analogue, see: Shi (2009 ▶).
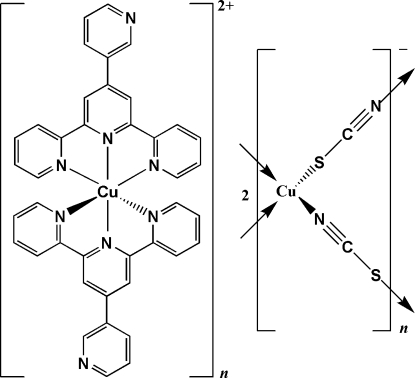


## Experimental

### 

#### Crystal data

[Cu(C_20_H_14_N_4_)_2_][Cu_2_(NCS)_4_]
*M* *_r_* = 1043.64
Triclinic, 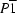

*a* = 10.0031 (6) Å
*b* = 10.2202 (6) Å
*c* = 21.2612 (12) Å
α = 82.607 (1)°
β = 87.732 (1)°
γ = 80.132 (1)°
*V* = 2123.3 (2) Å^3^
*Z* = 2
Mo *K*α radiation
μ = 1.74 mm^−1^
*T* = 295 K
0.16 × 0.13 × 0.11 mm

#### Data collection

Bruker SMART APEX area-detector diffractometer
Absorption correction: multi-scan (*SADABS*; Sheldrick, 1996 ▶) *T*_min_ = 0.769, *T*_max_ = 0.832
16747 measured reflections
8252 independent reflections
6751 reflections with *I* > 2σ(*I*)
*R*_int_ = 0.022

#### Refinement

*R*[*F* ^2^ > 2σ(*F* ^2^)] = 0.044
*wR*(*F* ^2^) = 0.103
*S* = 1.03
8252 reflections
568 parameters
H-atom parameters constrained
Δρ_max_ = 0.69 e Å^−3^
Δρ_min_ = −0.50 e Å^−3^

### 

Data collection: *SMART* (Bruker, 2002 ▶); cell refinement: *SAINT* (Bruker, 2002 ▶); data reduction: *SAINT*; program(s) used to solve structure: *SHELXS97* (Sheldrick, 2008 ▶); program(s) used to refine structure: *SHELXL97* (Sheldrick, 2008 ▶); molecular graphics: *SHELXTL* (Sheldrick, 2008 ▶); software used to prepare material for publication: *SHELXTL*.

## Supplementary Material

Crystal structure: contains datablocks I, global. DOI: 10.1107/S1600536809023356/zl2221sup1.cif

Structure factors: contains datablocks I. DOI: 10.1107/S1600536809023356/zl2221Isup2.hkl

Additional supplementary materials:  crystallographic information; 3D view; checkCIF report

## Figures and Tables

**Table 1:** Hydrogen-bond geometry (Å, °)

*D*—H⋯*A*	*D*—H	H⋯*A*	*D*⋯*A*	*D*—H⋯*A*
C4—H4⋯S4^i^	0.93	2.87	3.756 (3)	160
C15—H15⋯S2^ii^	0.93	2.83	3.676 (4)	151
C17—H17⋯S4^i^	0.93	2.80	3.650 (3)	152
C21—H21⋯S4^iii^	0.93	2.79	3.627 (3)	150
C29—H29⋯S2^iv^	0.93	2.78	3.654 (3)	156
C35—H35⋯N4^v^	0.93	2.47	3.217 (4)	137

Symmetry codes: (i) 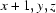; (ii) 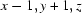; (iii) 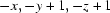; (iv) 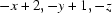; (v) 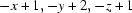.

**Fig. 1.**
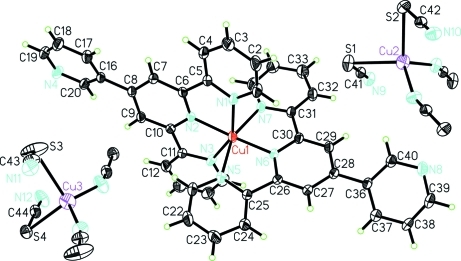
The title compound with displacement ellipsoids drawn at the 30% probability level.

**Fig. 2.**
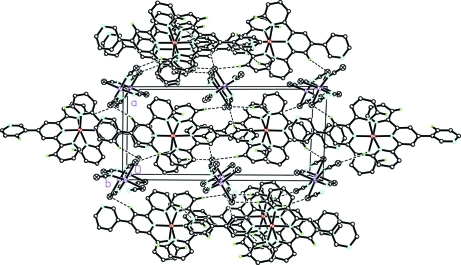
A packing diagram of the title compound, showing the intermolecular C–H···N and C–H···S hydrogen bonds as dashed lines. The H atoms not involved in hydrogen bonds have been omitted for clarity.

## supplementary crystallographic information

### Comment

2,2':6',2''-Terpyridine and its derivatives have been intensively explored because of the interesting electronic, photonic, magnetic, reactive and structural properties shown by the transition metal complexes of these ligands (Andres & Schubert, 2004; Constable, 1986; Hofmeier & Schubert, 2004). We report here the synthesis and structure of the Cu^II^ complex based on the 4'-(3-pyridyl)-2,2':6',2''-terpyridine (3-pytpy) ligand.

Fig. 1 illustrates the essential structural features of the title complex which consists of a packing of one [Cu(3-pytpy)_2_]^2+^ cation with two independent crystallographically centrosymmetric polymeric [Cu(SCN)_2_]^-^ anions. The central Cu^II^ ion in the cation is coordinated by two tridentate chelating units of the two 3-pytpy ligands to form an octahedral coordination geometry. Each Cu^I^ ion in the anion exhibits a distorted tetrahedral geometry and is coordinated by two S atoms and two N atoms from four thiocyanate ligands. Each thiocyanate ligand acts as a 1,3-µ_2_ bridge to link two Cu^I^ ions to generate two isostructural [Cu(SCN)_2_]_n_^n-^ anionic chains. The terpyridyl units of the 3-pytpy ligands are approximately planar [interannular torsion angles: 3.7 (1) °, 10.3 (2) °; 7.9 (3) °, 8.6 (2) °], the dihedral angles between the pendant and central pyridine ring are 18.4 (1) ° and 38.1 (2) °, respectively.

In the crystal packing, the neighbouring cationic units are packed by intermolecular C–H···N hydrogen bonds, and the [Cu(SCN)_2_]_n_^n-^ anionic chains are involved in intermolecular C–H···S hydrogen bonding interactions with the –CH groups of the 3-pytpy ligands, resulting in a three-dimensional supramolecular structure (Fig. 2).

### Experimental

A mixture of copper acetate hydrate (40.1 mg, 0.20 mmol), 3-pytpy (31.0 mg, 0.10 mmol) and ammonium thiocyanate (15.3 mg, 0.20 mmol) in ethanol (10 ml) was sealed in a 15 ml Teflon-lined reactor, heated to 418 K for 72 h, and then cooled to room temperature at a rate of 6 K/h to give black crystals of the title compound. Yield: 9 mg (17%).

### Refinement

The carbon-bound H atoms were placed at calculated positions (C—H = 0.93 Å) and refined as riding, with *U*_iso_(H) = 1.2*U*_eq_(C).

### Crystal data

**Table:**

[Cu(C_20_H_14_N_4_)_2_][Cu_2_(NCS)_4_]	*Z* = 2
*M**_r_* = 1043.64	*F*(000) = 1054
Triclinic, *P*1	*D*_x_ = 1.632 Mg m^−^^3^
Hall symbol: -P 1	Mo *K*α radiation, λ = 0.71073 Å
*a* = 10.0031 (6) Å	Cell parameters from 4926 reflections
*b* = 10.2202 (6) Å	θ = 2.4–25.2°
*c* = 21.2612 (12) Å	µ = 1.74 mm^−^^1^
α = 82.607 (1)°	*T* = 295 K
β = 87.732 (1)°	Block, black
γ = 80.132 (1)°	0.16 × 0.13 × 0.11 mm
*V* = 2123.3 (2) Å^3^	

### Data collection

**Table:**

Bruker SMART APEX area-detector diffractometer	8252 independent reflections
Radiation source: fine-focus sealed tube	6751 reflections with *I* > 2σ(*I*)
graphite	*R*_int_ = 0.022
φ and ω scans	θ_max_ = 26.0°, θ_min_ = 2.0°
Absorption correction: multi-scan (*SADABS*; Sheldrick, 1996)	*h* = −12→12
*T*_min_ = 0.769, *T*_max_ = 0.832	*k* = −12→12
16747 measured reflections	*l* = −26→26

### Refinement

**Table:**

Refinement on *F*^2^	Primary atom site location: structure-invariant direct methods
Least-squares matrix: full	Secondary atom site location: difference Fourier map
*R*[*F*^2^ > 2σ(*F*^2^)] = 0.044	Hydrogen site location: inferred from neighbouring sites
*wR*(*F*^2^) = 0.103	H-atom parameters constrained
*S* = 1.03	*w* = 1/[σ^2^(*F*_o_^2^) + (0.0476*P*)^2^ + 1.2038*P*] where *P* = (*F*_o_^2^ + 2*F*_c_^2^)/3
8252 reflections	(Δ/σ)_max_ = 0.001
568 parameters	Δρ_max_ = 0.69 e Å^−^^3^
0 restraints	Δρ_min_ = −0.50 e Å^−^^3^

### Special details

**Table:**

Geometry. All e.s.d.'s (except the e.s.d. in the dihedral angle between two l.s. planes) are estimated using the full covariance matrix. The cell e.s.d.'s are taken into account individually in the estimation of e.s.d.'s in distances, angles and torsion angles; correlations between e.s.d.'s in cell parameters are only used when they are defined by crystal symmetry. An approximate (isotropic) treatment of cell e.s.d.'s is used for estimating e.s.d.'s involving l.s. planes.
Refinement. Refinement of *F*^2^ against ALL reflections. The weighted *R*-factor *wR* and goodness of fit *S* are based on *F*^2^, conventional *R*-factors *R* are based on *F*, with *F* set to zero for negative *F*^2^. The threshold expression of *F*^2^ > σ(*F*^2^) is used only for calculating *R*-factors(gt) *etc*. and is not relevant to the choice of reflections for refinement. *R*-factors based on *F*^2^ are statistically about twice as large as those based on *F*, and *R*- factors based on ALL data will be even larger.

### Fractional atomic coordinates and isotropic or equivalent isotropic displacement parameters (Å^2^)

**Table:**

	*x*	*y*	*z*	*U*_iso_*/*U*_eq_	
Cu1	0.47293 (4)	0.80579 (4)	0.251577 (17)	0.03901 (11)	
Cu2	1.01294 (4)	0.24421 (4)	0.02065 (2)	0.04908 (13)	
Cu3	−0.01615 (5)	0.25397 (4)	0.48921 (2)	0.05715 (14)	
N1	0.6206 (3)	0.6156 (3)	0.28018 (11)	0.0387 (6)	
N2	0.4788 (2)	0.7971 (2)	0.34566 (10)	0.0327 (5)	
N3	0.3369 (3)	0.9916 (3)	0.27154 (12)	0.0465 (7)	
N4	0.4223 (3)	0.8771 (3)	0.64121 (12)	0.0518 (7)	
N5	0.2940 (3)	0.7189 (3)	0.24240 (11)	0.0437 (6)	
N6	0.4761 (2)	0.7898 (2)	0.16064 (10)	0.0321 (5)	
N7	0.6464 (3)	0.8943 (3)	0.21930 (11)	0.0381 (6)	
N8	0.6683 (3)	0.6192 (3)	−0.11272 (13)	0.0522 (7)	
N9	0.9311 (3)	0.6200 (3)	0.04686 (12)	0.0467 (7)	
N10	1.1099 (3)	−0.1402 (3)	0.01414 (14)	0.0470 (7)	
N11	0.0763 (3)	−0.1208 (3)	0.55590 (14)	0.0511 (7)	
N12	−0.0855 (3)	0.6058 (3)	0.55087 (16)	0.0623 (9)	
C1	0.6908 (4)	0.5291 (3)	0.24430 (15)	0.0472 (8)	
H1	0.6739	0.5408	0.2011	0.057*	
C2	0.7870 (4)	0.4236 (4)	0.26798 (17)	0.0572 (10)	
H2	0.8322	0.3635	0.2417	0.069*	
C3	0.8147 (4)	0.4092 (4)	0.33145 (18)	0.0645 (11)	
H3	0.8793	0.3388	0.3489	0.077*	
C4	0.7461 (3)	0.4997 (4)	0.36902 (15)	0.0548 (10)	
H4	0.7652	0.4922	0.4119	0.066*	
C5	0.6487 (3)	0.6016 (3)	0.34224 (13)	0.0368 (7)	
C6	0.5626 (3)	0.6988 (3)	0.37951 (13)	0.0339 (6)	
C7	0.5660 (3)	0.6887 (3)	0.44499 (13)	0.0354 (7)	
H7	0.6278	0.6222	0.4673	0.042*	
C8	0.4769 (3)	0.7781 (3)	0.47749 (13)	0.0325 (6)	
C9	0.3883 (3)	0.8766 (3)	0.44160 (13)	0.0348 (6)	
H9	0.3249	0.9360	0.4616	0.042*	
C10	0.3944 (3)	0.8864 (3)	0.37578 (13)	0.0338 (6)	
C11	0.3129 (3)	0.9965 (3)	0.33412 (14)	0.0388 (7)	
C12	0.2258 (3)	1.1015 (4)	0.35601 (17)	0.0586 (10)	
H12	0.2097	1.1030	0.3993	0.070*	
C13	0.1628 (4)	1.2041 (5)	0.3131 (2)	0.0748 (13)	
H13	0.1053	1.2764	0.3272	0.090*	
C14	0.1857 (4)	1.1985 (5)	0.24968 (19)	0.0760 (13)	
H14	0.1433	1.2659	0.2199	0.091*	
C15	0.2728 (4)	1.0909 (5)	0.23085 (17)	0.0650 (11)	
H15	0.2878	1.0870	0.1876	0.078*	
C16	0.4781 (3)	0.7684 (3)	0.54771 (13)	0.0333 (6)	
C17	0.5365 (3)	0.6531 (3)	0.58521 (14)	0.0449 (8)	
H17	0.5751	0.5774	0.5668	0.054*	
C18	0.5366 (4)	0.6524 (4)	0.64972 (16)	0.0528 (9)	
H18	0.5758	0.5761	0.6755	0.063*	
C19	0.4787 (4)	0.7644 (4)	0.67586 (15)	0.0521 (9)	
H19	0.4787	0.7621	0.7197	0.063*	
C20	0.4238 (3)	0.8768 (3)	0.57879 (14)	0.0412 (7)	
H20	0.3855	0.9552	0.5542	0.049*	
C21	0.1992 (4)	0.6952 (4)	0.28617 (16)	0.0595 (10)	
H21	0.2133	0.7078	0.3278	0.071*	
C22	0.0817 (4)	0.6531 (5)	0.27245 (18)	0.0662 (11)	
H22	0.0180	0.6368	0.3043	0.079*	
C23	0.0595 (4)	0.6354 (5)	0.21107 (19)	0.0643 (11)	
H23	−0.0191	0.6064	0.2007	0.077*	
C24	0.1557 (3)	0.6613 (4)	0.16508 (16)	0.0478 (8)	
H24	0.1421	0.6515	0.1230	0.057*	
C25	0.2718 (3)	0.7017 (3)	0.18210 (13)	0.0344 (6)	
C26	0.3818 (3)	0.7318 (3)	0.13663 (13)	0.0312 (6)	
C27	0.3933 (3)	0.7013 (3)	0.07485 (13)	0.0339 (6)	
H27	0.3275	0.6613	0.0587	0.041*	
C28	0.5031 (3)	0.7306 (3)	0.03723 (13)	0.0353 (7)	
C29	0.5989 (3)	0.7911 (3)	0.06305 (13)	0.0386 (7)	
H29	0.6735	0.8120	0.0387	0.046*	
C30	0.5832 (3)	0.8204 (3)	0.12494 (13)	0.0336 (6)	
C31	0.6784 (3)	0.8833 (3)	0.15784 (13)	0.0352 (7)	
C32	0.7917 (3)	0.9271 (4)	0.12923 (15)	0.0494 (8)	
H32	0.8125	0.9182	0.0868	0.059*	
C33	0.8734 (4)	0.9842 (4)	0.16454 (18)	0.0600 (10)	
H33	0.9493	1.0159	0.1460	0.072*	
C34	0.8420 (4)	0.9938 (4)	0.22712 (18)	0.0588 (10)	
H34	0.8969	1.0308	0.2518	0.071*	
C35	0.7277 (4)	0.9480 (4)	0.25305 (16)	0.0506 (9)	
H35	0.7065	0.9547	0.2956	0.061*	
C36	0.5201 (3)	0.6971 (3)	−0.02901 (13)	0.0369 (7)	
C37	0.4111 (4)	0.7106 (4)	−0.06815 (16)	0.0576 (10)	
H37	0.3239	0.7406	−0.0534	0.069*	
C38	0.4316 (4)	0.6796 (4)	−0.12910 (16)	0.0629 (11)	
H38	0.3589	0.6894	−0.1563	0.075*	
C39	0.5585 (4)	0.6349 (4)	−0.14877 (15)	0.0538 (9)	
H39	0.5709	0.6135	−0.1900	0.065*	
C40	0.6475 (3)	0.6510 (3)	−0.05347 (14)	0.0416 (7)	
H40	0.7221	0.6416	−0.0275	0.050*	
C41	0.9319 (3)	0.5134 (3)	0.07233 (14)	0.0372 (7)	
C42	1.1444 (3)	−0.0539 (3)	0.03533 (14)	0.0360 (7)	
C43	0.0940 (3)	−0.0174 (4)	0.56498 (16)	0.0481 (8)	
C44	−0.1332 (3)	0.5119 (3)	0.54818 (15)	0.0424 (7)	
S1	0.93354 (10)	0.35967 (9)	0.10770 (4)	0.0526 (2)	
S2	1.19304 (8)	0.07079 (8)	0.06543 (4)	0.04238 (19)	
S3	0.12114 (14)	0.13226 (10)	0.57617 (7)	0.0930 (5)	
S4	−0.20423 (8)	0.38015 (8)	0.54243 (4)	0.0432 (2)	

### Atomic displacement parameters (Å^2^)

**Table:**

	*U*^11^	*U*^22^	*U*^33^	*U*^12^	*U*^13^	*U*^23^
Cu1	0.0398 (2)	0.0517 (2)	0.0291 (2)	−0.01341 (18)	0.00678 (15)	−0.01289 (16)
Cu2	0.0598 (3)	0.0413 (2)	0.0492 (3)	−0.0138 (2)	−0.0012 (2)	−0.01005 (19)
Cu3	0.0643 (3)	0.0389 (2)	0.0721 (3)	−0.0152 (2)	−0.0013 (2)	−0.0129 (2)
N1	0.0449 (15)	0.0456 (15)	0.0255 (12)	−0.0068 (12)	0.0033 (11)	−0.0066 (11)
N2	0.0296 (12)	0.0454 (15)	0.0251 (12)	−0.0077 (11)	0.0023 (10)	−0.0105 (10)
N3	0.0477 (16)	0.0646 (19)	0.0276 (13)	−0.0092 (14)	−0.0033 (12)	−0.0070 (13)
N4	0.0620 (19)	0.0620 (19)	0.0304 (14)	0.0020 (15)	−0.0043 (13)	−0.0172 (13)
N5	0.0398 (15)	0.0644 (18)	0.0284 (13)	−0.0121 (13)	0.0027 (11)	−0.0082 (12)
N6	0.0341 (13)	0.0393 (14)	0.0231 (12)	−0.0071 (11)	0.0005 (10)	−0.0036 (10)
N7	0.0422 (14)	0.0476 (15)	0.0273 (12)	−0.0108 (12)	0.0052 (11)	−0.0128 (11)
N8	0.0590 (18)	0.0608 (19)	0.0391 (16)	−0.0101 (15)	0.0085 (14)	−0.0171 (14)
N9	0.0602 (18)	0.0456 (17)	0.0354 (15)	−0.0108 (14)	0.0009 (13)	−0.0079 (13)
N10	0.0471 (16)	0.0381 (15)	0.0561 (18)	−0.0028 (13)	−0.0121 (13)	−0.0085 (13)
N11	0.0586 (18)	0.0425 (17)	0.0542 (18)	−0.0103 (14)	−0.0072 (14)	−0.0089 (14)
N12	0.061 (2)	0.0516 (19)	0.081 (2)	−0.0240 (16)	0.0248 (17)	−0.0224 (17)
C1	0.060 (2)	0.054 (2)	0.0272 (16)	−0.0068 (17)	0.0052 (15)	−0.0116 (15)
C2	0.063 (2)	0.061 (2)	0.042 (2)	0.0087 (19)	0.0159 (17)	−0.0168 (17)
C3	0.054 (2)	0.078 (3)	0.048 (2)	0.023 (2)	0.0072 (17)	−0.0057 (19)
C4	0.047 (2)	0.082 (3)	0.0281 (16)	0.0123 (19)	0.0019 (14)	−0.0086 (17)
C5	0.0351 (16)	0.0483 (18)	0.0268 (15)	−0.0039 (14)	0.0038 (12)	−0.0087 (13)
C6	0.0292 (15)	0.0465 (18)	0.0283 (15)	−0.0093 (13)	0.0024 (12)	−0.0105 (13)
C7	0.0356 (16)	0.0430 (17)	0.0268 (14)	−0.0033 (13)	0.0009 (12)	−0.0064 (13)
C8	0.0345 (15)	0.0404 (16)	0.0255 (14)	−0.0135 (13)	0.0044 (12)	−0.0072 (12)
C9	0.0363 (16)	0.0402 (16)	0.0290 (15)	−0.0070 (13)	0.0064 (12)	−0.0092 (13)
C10	0.0301 (15)	0.0438 (17)	0.0292 (15)	−0.0087 (13)	0.0000 (12)	−0.0071 (13)
C11	0.0330 (16)	0.055 (2)	0.0283 (15)	−0.0072 (14)	0.0004 (12)	−0.0051 (14)
C12	0.044 (2)	0.080 (3)	0.0402 (19)	0.0129 (19)	0.0069 (15)	−0.0007 (18)
C13	0.055 (2)	0.092 (3)	0.061 (3)	0.024 (2)	0.001 (2)	0.008 (2)
C14	0.059 (2)	0.103 (4)	0.050 (2)	0.015 (2)	−0.0102 (19)	0.016 (2)
C15	0.064 (3)	0.096 (3)	0.0342 (19)	−0.014 (2)	−0.0097 (17)	0.000 (2)
C16	0.0346 (16)	0.0427 (17)	0.0242 (14)	−0.0102 (13)	0.0035 (12)	−0.0061 (12)
C17	0.053 (2)	0.0462 (19)	0.0344 (17)	−0.0043 (16)	0.0068 (14)	−0.0074 (14)
C18	0.061 (2)	0.058 (2)	0.0348 (18)	−0.0044 (18)	−0.0021 (16)	0.0047 (16)
C19	0.057 (2)	0.075 (3)	0.0257 (16)	−0.0123 (19)	−0.0019 (15)	−0.0062 (17)
C20	0.0469 (18)	0.0463 (18)	0.0289 (15)	−0.0011 (15)	−0.0035 (13)	−0.0070 (13)
C21	0.055 (2)	0.098 (3)	0.0286 (17)	−0.022 (2)	0.0074 (15)	−0.0111 (18)
C22	0.059 (2)	0.099 (3)	0.045 (2)	−0.029 (2)	0.0206 (18)	−0.009 (2)
C23	0.045 (2)	0.097 (3)	0.060 (2)	−0.032 (2)	0.0101 (18)	−0.020 (2)
C24	0.0423 (18)	0.068 (2)	0.0371 (17)	−0.0172 (17)	0.0024 (14)	−0.0133 (16)
C25	0.0338 (15)	0.0404 (17)	0.0282 (15)	−0.0036 (13)	0.0000 (12)	−0.0053 (12)
C26	0.0297 (14)	0.0363 (16)	0.0261 (14)	−0.0032 (12)	−0.0029 (11)	−0.0015 (12)
C27	0.0355 (16)	0.0396 (16)	0.0273 (14)	−0.0066 (13)	−0.0033 (12)	−0.0055 (12)
C28	0.0386 (16)	0.0414 (17)	0.0254 (14)	−0.0047 (13)	−0.0023 (12)	−0.0046 (12)
C29	0.0408 (17)	0.0500 (19)	0.0274 (15)	−0.0151 (15)	0.0058 (13)	−0.0057 (13)
C30	0.0377 (16)	0.0385 (16)	0.0247 (14)	−0.0092 (13)	0.0016 (12)	−0.0010 (12)
C31	0.0395 (16)	0.0406 (17)	0.0270 (14)	−0.0097 (13)	0.0029 (12)	−0.0069 (12)
C32	0.053 (2)	0.068 (2)	0.0335 (17)	−0.0271 (18)	0.0107 (15)	−0.0125 (16)
C33	0.056 (2)	0.080 (3)	0.056 (2)	−0.037 (2)	0.0086 (18)	−0.019 (2)
C34	0.061 (2)	0.073 (3)	0.054 (2)	−0.027 (2)	−0.0038 (18)	−0.0269 (19)
C35	0.057 (2)	0.064 (2)	0.0358 (18)	−0.0140 (18)	0.0024 (15)	−0.0226 (16)
C36	0.0420 (17)	0.0445 (18)	0.0249 (14)	−0.0087 (14)	0.0001 (12)	−0.0056 (13)
C37	0.045 (2)	0.088 (3)	0.0385 (19)	−0.0023 (19)	−0.0017 (15)	−0.0151 (18)
C38	0.055 (2)	0.104 (3)	0.0302 (18)	−0.007 (2)	−0.0104 (16)	−0.0191 (19)
C39	0.069 (3)	0.066 (2)	0.0287 (17)	−0.012 (2)	0.0001 (16)	−0.0155 (16)
C40	0.0486 (19)	0.0453 (18)	0.0334 (16)	−0.0105 (15)	0.0000 (14)	−0.0104 (14)
C41	0.0356 (16)	0.050 (2)	0.0283 (15)	−0.0066 (14)	−0.0003 (12)	−0.0138 (14)
C42	0.0324 (16)	0.0390 (17)	0.0332 (16)	0.0005 (13)	−0.0025 (12)	0.0003 (13)
C43	0.0436 (19)	0.047 (2)	0.053 (2)	0.0014 (16)	−0.0157 (16)	−0.0117 (16)
C44	0.0419 (18)	0.0455 (19)	0.0380 (17)	−0.0054 (15)	0.0085 (14)	−0.0040 (14)
S1	0.0757 (6)	0.0453 (5)	0.0377 (5)	−0.0136 (4)	0.0099 (4)	−0.0066 (4)
S2	0.0480 (5)	0.0433 (4)	0.0378 (4)	−0.0120 (4)	−0.0076 (3)	−0.0045 (3)
S3	0.1096 (10)	0.0421 (6)	0.1313 (11)	0.0040 (6)	−0.0765 (9)	−0.0253 (6)
S4	0.0447 (5)	0.0419 (4)	0.0439 (5)	−0.0118 (4)	0.0051 (4)	−0.0041 (4)

### Geometric parameters (Å, °)

**Table:**

Cu1—N6	1.960 (2)	C10—C11	1.484 (4)
Cu1—N2	1.994 (2)	C11—C12	1.380 (5)
Cu1—N7	2.141 (3)	C12—C13	1.379 (5)
Cu1—N5	2.156 (3)	C12—H12	0.9300
Cu1—N3	2.217 (3)	C13—C14	1.366 (6)
Cu1—N1	2.261 (3)	C13—H13	0.9300
Cu2—N10^i^	1.977 (3)	C14—C15	1.373 (6)
Cu2—N9^ii^	1.997 (3)	C14—H14	0.9300
Cu2—S1	2.3607 (10)	C15—H15	0.9300
Cu2—S2	2.4291 (9)	C16—C20	1.383 (4)
Cu3—N11^iii^	1.948 (3)	C16—C17	1.387 (4)
Cu3—N12^iv^	1.983 (3)	C17—C18	1.371 (4)
Cu3—S4	2.4204 (10)	C17—H17	0.9300
Cu3—S3	2.4239 (12)	C18—C19	1.366 (5)
N1—C1	1.333 (4)	C18—H18	0.9300
N1—C5	1.345 (4)	C19—H19	0.9300
N2—C10	1.341 (4)	C20—H20	0.9300
N2—C6	1.343 (4)	C21—C22	1.373 (5)
N3—C15	1.336 (5)	C21—H21	0.9300
N3—C11	1.348 (4)	C22—C23	1.372 (5)
N4—C20	1.327 (4)	C22—H22	0.9300
N4—C19	1.334 (5)	C23—C24	1.378 (5)
N5—C21	1.332 (4)	C23—H23	0.9300
N5—C25	1.348 (4)	C24—C25	1.374 (4)
N6—C26	1.345 (3)	C24—H24	0.9300
N6—C30	1.345 (4)	C25—C26	1.482 (4)
N7—C35	1.334 (4)	C26—C27	1.385 (4)
N7—C31	1.347 (3)	C27—C28	1.385 (4)
N8—C39	1.340 (4)	C27—H27	0.9300
N8—C40	1.341 (4)	C28—C29	1.391 (4)
N9—C41	1.152 (4)	C28—C36	1.488 (4)
N9—Cu2^ii^	1.997 (3)	C29—C30	1.383 (4)
N10—C42	1.149 (4)	C29—H29	0.9300
N10—Cu2^i^	1.977 (3)	C30—C31	1.480 (4)
N11—C43	1.142 (4)	C31—C32	1.379 (4)
N11—Cu3^iii^	1.948 (3)	C32—C33	1.378 (5)
N12—C44	1.151 (4)	C32—H32	0.9300
N12—Cu3^iv^	1.983 (3)	C33—C34	1.367 (5)
C1—C2	1.373 (5)	C33—H33	0.9300
C1—H1	0.9300	C34—C35	1.377 (5)
C2—C3	1.373 (5)	C34—H34	0.9300
C2—H2	0.9300	C35—H35	0.9300
C3—C4	1.376 (5)	C36—C37	1.375 (4)
C3—H3	0.9300	C36—C40	1.386 (4)
C4—C5	1.379 (4)	C37—C38	1.372 (5)
C4—H4	0.9300	C37—H37	0.9300
C5—C6	1.487 (4)	C38—C39	1.343 (5)
C6—C7	1.384 (4)	C38—H38	0.9300
C7—C8	1.393 (4)	C39—H39	0.9300
C7—H7	0.9300	C40—H40	0.9300
C8—C9	1.389 (4)	C41—S1	1.650 (4)
C8—C16	1.484 (4)	C42—S2	1.648 (3)
C9—C10	1.390 (4)	C43—S3	1.647 (4)
C9—H9	0.9300	C44—S4	1.647 (4)
			
N6—Cu1—N2	172.70 (10)	C13—C14—C15	118.5 (4)
N6—Cu1—N7	78.36 (9)	C13—C14—H14	120.7
N2—Cu1—N7	103.42 (9)	C15—C14—H14	120.7
N6—Cu1—N5	78.09 (9)	N3—C15—C14	123.2 (4)
N2—Cu1—N5	100.30 (9)	N3—C15—H15	118.4
N7—Cu1—N5	156.29 (9)	C14—C15—H15	118.4
N6—Cu1—N3	110.24 (10)	C20—C16—C17	116.8 (3)
N2—Cu1—N3	76.69 (10)	C20—C16—C8	120.9 (3)
N7—Cu1—N3	97.67 (10)	C17—C16—C8	122.3 (3)
N5—Cu1—N3	87.92 (10)	C18—C17—C16	119.1 (3)
N6—Cu1—N1	96.53 (9)	C18—C17—H17	120.4
N2—Cu1—N1	76.60 (9)	C16—C17—H17	120.4
N7—Cu1—N1	86.65 (10)	C19—C18—C17	119.6 (3)
N5—Cu1—N1	98.71 (10)	C19—C18—H18	120.2
N3—Cu1—N1	153.21 (9)	C17—C18—H18	120.2
N10^i^—Cu2—N9^ii^	110.41 (11)	N4—C19—C18	122.9 (3)
N10^i^—Cu2—S1	117.89 (9)	N4—C19—H19	118.6
N9^ii^—Cu2—S1	106.52 (8)	C18—C19—H19	118.6
N10^i^—Cu2—S2	102.63 (8)	N4—C20—C16	124.7 (3)
N9^ii^—Cu2—S2	116.21 (9)	N4—C20—H20	117.6
S1—Cu2—S2	103.41 (3)	C16—C20—H20	117.6
N11^iii^—Cu3—N12^iv^	124.29 (13)	N5—C21—C22	122.8 (3)
N11^iii^—Cu3—S4	111.33 (9)	N5—C21—H21	118.6
N12^iv^—Cu3—S4	103.13 (9)	C22—C21—H21	118.6
N11^iii^—Cu3—S3	106.80 (9)	C23—C22—C21	119.0 (3)
N12^iv^—Cu3—S3	106.13 (11)	C23—C22—H22	120.5
S4—Cu3—S3	103.22 (5)	C21—C22—H22	120.5
C1—N1—C5	118.3 (3)	C22—C23—C24	118.9 (3)
C1—N1—Cu1	129.8 (2)	C22—C23—H23	120.6
C5—N1—Cu1	111.40 (19)	C24—C23—H23	120.6
C10—N2—C6	119.6 (2)	C25—C24—C23	119.2 (3)
C10—N2—Cu1	119.94 (19)	C25—C24—H24	120.4
C6—N2—Cu1	120.44 (19)	C23—C24—H24	120.4
C15—N3—C11	118.2 (3)	N5—C25—C24	122.1 (3)
C15—N3—Cu1	129.1 (2)	N5—C25—C26	114.2 (3)
C11—N3—Cu1	112.6 (2)	C24—C25—C26	123.7 (3)
C20—N4—C19	116.9 (3)	N6—C26—C27	120.8 (3)
C21—N5—C25	118.0 (3)	N6—C26—C25	114.3 (2)
C21—N5—Cu1	128.6 (2)	C27—C26—C25	124.9 (3)
C25—N5—Cu1	112.98 (19)	C26—C27—C28	119.8 (3)
C26—N6—C30	120.7 (2)	C26—C27—H27	120.1
C26—N6—Cu1	119.71 (18)	C28—C27—H27	120.1
C30—N6—Cu1	119.00 (19)	C27—C28—C29	118.4 (3)
C35—N7—C31	118.6 (3)	C27—C28—C36	121.3 (3)
C35—N7—Cu1	128.2 (2)	C29—C28—C36	120.4 (3)
C31—N7—Cu1	112.99 (19)	C30—C29—C28	120.0 (3)
C39—N8—C40	116.7 (3)	C30—C29—H29	120.0
C41—N9—Cu2^ii^	154.6 (3)	C28—C29—H29	120.0
C42—N10—Cu2^i^	159.1 (3)	N6—C30—C29	120.5 (3)
C43—N11—Cu3^iii^	157.5 (3)	N6—C30—C31	114.5 (2)
C44—N12—Cu3^iv^	150.6 (3)	C29—C30—C31	125.0 (3)
N1—C1—C2	123.3 (3)	N7—C31—C32	121.7 (3)
N1—C1—H1	118.4	N7—C31—C30	114.4 (2)
C2—C1—H1	118.4	C32—C31—C30	123.9 (3)
C3—C2—C1	118.1 (3)	C33—C32—C31	118.9 (3)
C3—C2—H2	120.9	C33—C32—H32	120.5
C1—C2—H2	120.9	C31—C32—H32	120.5
C2—C3—C4	119.5 (3)	C34—C33—C32	119.4 (3)
C2—C3—H3	120.2	C34—C33—H33	120.3
C4—C3—H3	120.2	C32—C33—H33	120.3
C3—C4—C5	119.1 (3)	C33—C34—C35	119.0 (3)
C3—C4—H4	120.4	C33—C34—H34	120.5
C5—C4—H4	120.4	C35—C34—H34	120.5
N1—C5—C4	121.6 (3)	N7—C35—C34	122.4 (3)
N1—C5—C6	115.1 (3)	N7—C35—H35	118.8
C4—C5—C6	123.3 (3)	C34—C35—H35	118.8
N2—C6—C7	121.5 (3)	C37—C36—C40	117.6 (3)
N2—C6—C5	115.8 (2)	C37—C36—C28	121.7 (3)
C7—C6—C5	122.7 (3)	C40—C36—C28	120.8 (3)
C6—C7—C8	120.0 (3)	C38—C37—C36	119.7 (3)
C6—C7—H7	120.0	C38—C37—H37	120.1
C8—C7—H7	120.0	C36—C37—H37	120.1
C9—C8—C7	117.4 (3)	C39—C38—C37	118.7 (3)
C9—C8—C16	121.6 (3)	C39—C38—H38	120.6
C7—C8—C16	120.9 (3)	C37—C38—H38	120.6
C8—C9—C10	120.0 (3)	N8—C39—C38	124.2 (3)
C8—C9—H9	120.0	N8—C39—H39	117.9
C10—C9—H9	120.0	C38—C39—H39	117.9
N2—C10—C9	121.3 (3)	N8—C40—C36	123.1 (3)
N2—C10—C11	115.5 (2)	N8—C40—H40	118.4
C9—C10—C11	123.2 (3)	C36—C40—H40	118.4
N3—C11—C12	121.3 (3)	N9—C41—S1	179.0 (3)
N3—C11—C10	114.4 (3)	N10—C42—S2	179.6 (3)
C12—C11—C10	124.1 (3)	N11—C43—S3	178.5 (4)
C13—C12—C11	119.4 (3)	N12—C44—S4	178.2 (3)
C13—C12—H12	120.3	C41—S1—Cu2	97.99 (10)
C11—C12—H12	120.3	C42—S2—Cu2	96.50 (10)
C14—C13—C12	119.3 (4)	C43—S3—Cu3	96.18 (12)
C14—C13—H13	120.3	C44—S4—Cu3	97.22 (11)
C12—C13—H13	120.3		
			
N6—Cu1—N1—C1	3.8 (3)	C9—C10—C11—N3	177.4 (3)
N2—Cu1—N1—C1	−178.7 (3)	N2—C10—C11—C12	−176.5 (3)
N7—Cu1—N1—C1	−74.1 (3)	C9—C10—C11—C12	1.7 (5)
N5—Cu1—N1—C1	82.7 (3)	N3—C11—C12—C13	−0.5 (6)
N3—Cu1—N1—C1	−174.5 (3)	C10—C11—C12—C13	175.0 (4)
N6—Cu1—N1—C5	175.5 (2)	C11—C12—C13—C14	1.3 (7)
N2—Cu1—N1—C5	−7.0 (2)	C12—C13—C14—C15	−0.9 (7)
N7—Cu1—N1—C5	97.7 (2)	C11—N3—C15—C14	1.2 (6)
N5—Cu1—N1—C5	−105.6 (2)	Cu1—N3—C15—C14	176.9 (3)
N3—Cu1—N1—C5	−2.7 (3)	C13—C14—C15—N3	−0.4 (7)
N7—Cu1—N2—C10	103.0 (2)	C9—C8—C16—C20	−20.1 (4)
N5—Cu1—N2—C10	−77.2 (2)	C7—C8—C16—C20	159.5 (3)
N3—Cu1—N2—C10	8.2 (2)	C9—C8—C16—C17	161.9 (3)
N1—Cu1—N2—C10	−173.8 (2)	C7—C8—C16—C17	−18.5 (4)
N7—Cu1—N2—C6	−80.0 (2)	C20—C16—C17—C18	0.4 (5)
N5—Cu1—N2—C6	99.8 (2)	C8—C16—C17—C18	178.5 (3)
N3—Cu1—N2—C6	−174.8 (2)	C16—C17—C18—C19	0.4 (5)
N1—Cu1—N2—C6	3.2 (2)	C20—N4—C19—C18	0.2 (5)
N6—Cu1—N3—C15	−6.4 (3)	C17—C18—C19—N4	−0.7 (6)
N2—Cu1—N3—C15	176.0 (3)	C19—N4—C20—C16	0.7 (5)
N7—Cu1—N3—C15	73.9 (3)	C17—C16—C20—N4	−1.0 (5)
N5—Cu1—N3—C15	−82.9 (3)	C8—C16—C20—N4	−179.1 (3)
N1—Cu1—N3—C15	171.8 (3)	C25—N5—C21—C22	0.7 (6)
N6—Cu1—N3—C11	169.5 (2)	Cu1—N5—C21—C22	172.4 (3)
N2—Cu1—N3—C11	−8.1 (2)	N5—C21—C22—C23	−0.6 (7)
N7—Cu1—N3—C11	−110.2 (2)	C21—C22—C23—C24	−0.4 (7)
N5—Cu1—N3—C11	93.0 (2)	C22—C23—C24—C25	1.1 (6)
N1—Cu1—N3—C11	−12.3 (4)	C21—N5—C25—C24	0.1 (5)
N6—Cu1—N5—C21	−174.0 (3)	Cu1—N5—C25—C24	−172.9 (3)
N2—Cu1—N5—C21	13.3 (3)	C21—N5—C25—C26	179.3 (3)
N7—Cu1—N5—C21	−167.2 (3)	Cu1—N5—C25—C26	6.3 (3)
N3—Cu1—N5—C21	−62.8 (3)	C23—C24—C25—N5	−1.0 (5)
N1—Cu1—N5—C21	91.1 (3)	C23—C24—C25—C26	179.9 (3)
N6—Cu1—N5—C25	−1.9 (2)	C30—N6—C26—C27	0.6 (4)
N2—Cu1—N5—C25	−174.7 (2)	Cu1—N6—C26—C27	−170.3 (2)
N7—Cu1—N5—C25	4.8 (4)	C30—N6—C26—C25	178.7 (3)
N3—Cu1—N5—C25	109.3 (2)	Cu1—N6—C26—C25	7.9 (3)
N1—Cu1—N5—C25	−96.8 (2)	N5—C25—C26—N6	−9.2 (4)
N7—Cu1—N6—C26	179.3 (2)	C24—C25—C26—N6	170.0 (3)
N5—Cu1—N6—C26	−3.5 (2)	N5—C25—C26—C27	168.9 (3)
N3—Cu1—N6—C26	−86.7 (2)	C24—C25—C26—C27	−11.9 (5)
N1—Cu1—N6—C26	94.1 (2)	N6—C26—C27—C28	0.0 (4)
N7—Cu1—N6—C30	8.3 (2)	C25—C26—C27—C28	−178.0 (3)
N5—Cu1—N6—C30	−174.5 (2)	C26—C27—C28—C29	−0.3 (4)
N3—Cu1—N6—C30	102.3 (2)	C26—C27—C28—C36	178.9 (3)
N1—Cu1—N6—C30	−76.9 (2)	C27—C28—C29—C30	0.1 (5)
N6—Cu1—N7—C35	178.8 (3)	C36—C28—C29—C30	−179.2 (3)
N2—Cu1—N7—C35	−8.4 (3)	C26—N6—C30—C29	−0.8 (4)
N5—Cu1—N7—C35	172.0 (3)	Cu1—N6—C30—C29	170.1 (2)
N3—Cu1—N7—C35	69.6 (3)	C26—N6—C30—C31	−179.8 (2)
N1—Cu1—N7—C35	−83.8 (3)	Cu1—N6—C30—C31	−8.9 (3)
N6—Cu1—N7—C31	−6.2 (2)	C28—C29—C30—N6	0.5 (5)
N2—Cu1—N7—C31	166.6 (2)	C28—C29—C30—C31	179.4 (3)
N5—Cu1—N7—C31	−12.9 (4)	C35—N7—C31—C32	−0.6 (5)
N3—Cu1—N7—C31	−115.4 (2)	Cu1—N7—C31—C32	−176.1 (3)
N1—Cu1—N7—C31	91.2 (2)	C35—N7—C31—C30	179.0 (3)
C5—N1—C1—C2	2.4 (5)	Cu1—N7—C31—C30	3.5 (3)
Cu1—N1—C1—C2	173.7 (3)	N6—C30—C31—N7	3.0 (4)
N1—C1—C2—C3	−2.0 (6)	C29—C30—C31—N7	−176.0 (3)
C1—C2—C3—C4	0.0 (6)	N6—C30—C31—C32	−177.4 (3)
C2—C3—C4—C5	1.3 (6)	C29—C30—C31—C32	3.6 (5)
C1—N1—C5—C4	−0.9 (5)	N7—C31—C32—C33	−0.4 (5)
Cu1—N1—C5—C4	−173.7 (3)	C30—C31—C32—C33	−180.0 (3)
C1—N1—C5—C6	−177.9 (3)	C31—C32—C33—C34	1.2 (6)
Cu1—N1—C5—C6	9.3 (3)	C32—C33—C34—C35	−1.1 (6)
C3—C4—C5—N1	−0.9 (6)	C31—N7—C35—C34	0.8 (5)
C3—C4—C5—C6	175.7 (3)	Cu1—N7—C35—C34	175.5 (3)
C10—N2—C6—C7	−1.3 (4)	C33—C34—C35—N7	0.1 (6)
Cu1—N2—C6—C7	−178.3 (2)	C27—C28—C36—C37	38.3 (5)
C10—N2—C6—C5	177.8 (3)	C29—C28—C36—C37	−142.4 (3)
Cu1—N2—C6—C5	0.8 (3)	C27—C28—C36—C40	−141.7 (3)
N1—C5—C6—N2	−7.3 (4)	C29—C28—C36—C40	37.5 (4)
C4—C5—C6—N2	175.8 (3)	C40—C36—C37—C38	−0.5 (6)
N1—C5—C6—C7	171.8 (3)	C28—C36—C37—C38	179.4 (3)
C4—C5—C6—C7	−5.1 (5)	C36—C37—C38—C39	0.9 (6)
N2—C6—C7—C8	2.9 (4)	C40—N8—C39—C38	−0.1 (6)
C5—C6—C7—C8	−176.1 (3)	C37—C38—C39—N8	−0.6 (7)
C6—C7—C8—C9	−0.9 (4)	C39—N8—C40—C36	0.5 (5)
C6—C7—C8—C16	179.5 (3)	C37—C36—C40—N8	−0.2 (5)
C7—C8—C9—C10	−2.5 (4)	C28—C36—C40—N8	179.9 (3)
C16—C8—C9—C10	177.0 (3)	N10^i^—Cu2—S1—C41	−115.26 (14)
C6—N2—C10—C9	−2.3 (4)	N9^ii^—Cu2—S1—C41	9.39 (14)
Cu1—N2—C10—C9	174.7 (2)	S2—Cu2—S1—C41	132.37 (11)
C6—N2—C10—C11	175.9 (3)	N10^i^—Cu2—S2—C42	9.39 (14)
Cu1—N2—C10—C11	−7.0 (3)	N9^ii^—Cu2—S2—C42	−111.19 (14)
C8—C9—C10—N2	4.3 (4)	S1—Cu2—S2—C42	132.50 (11)
C8—C9—C10—C11	−173.8 (3)	N11^iii^—Cu3—S3—C43	−5.28 (17)
C15—N3—C11—C12	−0.7 (5)	N12^iv^—Cu3—S3—C43	−139.71 (17)
Cu1—N3—C11—C12	−177.1 (3)	S4—Cu3—S3—C43	112.18 (14)
C15—N3—C11—C10	−176.6 (3)	N11^iii^—Cu3—S4—C44	−158.24 (15)
Cu1—N3—C11—C10	7.0 (3)	N12^iv^—Cu3—S4—C44	−22.83 (16)
N2—C10—C11—N3	−0.8 (4)	S3—Cu3—S4—C44	87.53 (12)

Symmetry codes: (i) −*x*+2, −*y*, −*z*; (ii) −*x*+2, −*y*+1, −*z*; (iii) −*x*, −*y*, −*z*+1; (iv) −*x*, −*y*+1, −*z*+1.

### Hydrogen-bond geometry (Å, °)

**Table:**

*D*—H···*A*	*D*—H	H···*A*	*D*···*A*	*D*—H···*A*
C4—H4···S4^v^	0.93	2.87	3.756 (3)	160
C15—H15···S2^vi^	0.93	2.83	3.676 (4)	151
C17—H17···S4^v^	0.93	2.80	3.650 (3)	152
C21—H21···S4^iv^	0.93	2.79	3.627 (3)	150
C29—H29···S2^ii^	0.93	2.78	3.654 (3)	156
C35—H35···N4^vii^	0.93	2.47	3.217 (4)	137

Symmetry codes: (v) *x*+1, *y*, *z*; (vi) *x*−1, *y*+1, *z*; (iv) −*x*, −*y*+1, −*z*+1; (ii) −*x*+2, −*y*+1, −*z*; (vii) −*x*+1, −*y*+2, −*z*+1.
